# The bone marrow microenvironment as a mediator of chemoresistance in acute lymphoblastic leukemia

**DOI:** 10.20517/cdr.2019.63

**Published:** 2019-12-19

**Authors:** Lauren K. Meyer, Michelle L. Hermiston

**Affiliations:** Department of Pediatrics, University of California, San Francisco, SF 94158, USA.

**Keywords:** Acute lymphoblastic leukemia, chemotherapy, chemoresistance, bone marrow microenvironment

## Abstract

Acute lymphoblastic leukemia (ALL) is a malignancy of immature lymphoid cells that arises due to clonal expansion of cells that undergo developmental arrest and acquisition of pathogenic mutations. With the introduction of intensive multi-agent chemotherapeutic regimens, survival rates for ALL have improved dramatically over the past several decades, though survival rates for adult ALL continue to lag behind those of pediatric ALL. Resistance to chemotherapy remains a significant obstacle in the treatment of ALL, and chemoresistance due to molecular alterations within ALL cells have been described. In addition to these cell-intrinsic factors, the bone marrow microenvironment has more recently been appreciated as a cell-extrinsic mediator of chemoresistance, and it is now known that stromal cells within the bone marrow microenvironment, through direct cell-cell interactions and through the release of lymphoid-acting soluble factors, contribute to ALL pathogenesis and chemoresistance. This review discusses mechanisms of chemoresistance mediated by factors within the bone marrow microenvironment and highlights novel therapeutic strategies that have been investigated to overcome chemoresistance in this context.

## Introduction

Acute lymphoblastic leukemia (ALL) is a malignancy of immature lymphoid cells of either the B-cell (B-ALL) or T-cell (T-ALL) lineage. ALL is the most common malignancy of childhood, and survival rates for children with ALL now approach 90% on modern treatment protocols, with even better survival rates for some molecular subtypes^[1]^. In contrast, survival rates for adult ALL remain lower at 30%-40%^[2]^. In both contexts, improvements in survival were achieved in recent decades due in large part to the continued optimization of intensive multi-agent chemotherapy. The agents most commonly utilized in the treatment of ALL and their mechanisms of action are outlined in Table 1. Despite these improvements, patients with relapsed or refractory ALL continue to face inferior outcomes, underscoring the importance of studies aimed at understanding the drivers of chemoresistance and identifying novel therapeutic strategies to restore chemosensitivity.

**Table 1:** Mechanisms of action of chemotherapeutic agents used in the treatment of acute lymphoblastic leukemia

Drug	Mechanism of action
Asparaginase	Cleaves exogenous asparagine to deprive ALL cells of this amino acid
Clofarabine	Purine nucleoside analog that inhibits synthesis and repair of DNA
Corticosteroids (prednisone or dexamethasone)	Glucocorticoid receptor agonists that induce pro-apoptotic effects in lymphoid cells
Daunorubicin and Doxorubicin	Anthracyclines that inhibit topoisomerases
Etoposide	Topoisomerase II inhibitor
Methotrexate	Antimetabolite that inhibits dihydrofolate reductase to inhibit synthesis of purine nucleotides
Vincristine	Microtubule-destabilizing agent

The genomic landscapes of both pediatric and adult ALL have been extensively characterized^[3-5]^, and in many cases the presence of specific molecular or cytogenetic alterations can be correlated with prognosis or with resistance to a particular chemotherapeutic agent^[6-10]^. These genetic factors therefore represent cell-intrinsic drivers of chemoresistance, and there have been considerable efforts to target these resistance mechanisms through the use of biologic and small molecule therapeutic agents. Equally important however are the complex and diverse cell-extrinsic factors that similarly modulate response to chemotherapy and clinical outcomes in ALL.

Specifically, both normal hematopoietic progenitor cells and ALL cells residing in the bone marrow receive key survival, proliferative, and homing signals from the bone marrow microenvironment. This microenvironment is classically subdivided into two distinct niches comprised of the endosteal niche and the vascular niche. In the endosteal niche, hematopoietic cells maintain direct cell-cell interactions with osteoblasts, which support hematopoietic cell survival and proliferation. The vascular niche consists of small sinusoidal blood vessels within the marrow cavity, which ensure the movement of oxygen, nutrients, and cellular homing factors in and out of the bone marrow, thereby supporting hematopoietic cell function and maintaining communication with other tissue types^[11]^. Given these abundant interactions between the bone marrow microenvironment and ALL cells, there is significant interest in elucidating the microenvironmental drivers of chemoresistance in ALL and in identifying novel therapeutic strategies to overcome these cell-extrinsic mechanisms of chemoresistance^[12-14]^.

## Cell-cell interactions in the bone marrow microenvironment

A number of model systems of have been investigated for their ability to facilitate the *ex vivo* analysis of primary patient ALL cells, which demonstrate a high rate of spontaneous apoptosis under standard cell culture conditions. In particular, it has been shown that co-culture with bone marrow stromal cells (BMSCs) significantly enhances the survival of leukemic blasts *ex vivo*^[15]^. Specifically, this survival effect is often dependent upon direct contact with BMSCs, as BMSC conditioned media is insufficient to mediate the same improvement in cell survival^[16]^. These studies highlight the importance of cell-cell interactions with BMSCs for the survival and proliferation of ALL cells, and a number of studies, many of which are described below, have demonstrated that these cell-cell interactions mediate chemoresistance.

### Integrins

Integrins are a class of transmembrane cell adhesion receptors that make up a critical component of the extracellular matrix (ECM). Integrins are comprised of heterodimers of a number of distinct α and β subunits and each have specific ligand-binding capabilities, determined predominantly by the identity of the α subunit. Upon engagement by extracellular stimuli, activated integrins mediate a variety of signal transduction events leading to modulation of a wide range of cellular processes, including cell survival, gene expression, and cell motility^[17]^.

Despite the ubiquitous expression of this class of cell adhesion receptors throughout all tissue types, distinct integrin heterodimers demonstrate tissue-specific expression patterns. The β1-containing integrins, collectively known as very-late activation antigens (VLAs), heterodimerize with α4 subunits (CD49b) to form VLA-4. VLA-4 is highly expressed on hematopoietic progenitors and more mature blood cells, including healthy leukocytes and leukemic blasts, and functions to mediate trafficking of these cells within and out of the bone marrow^[17]^. Importantly, aberrant expression of VLA-4 in leukemic blasts has been associated with chemoresistance and poor clinical outcomes. For example, amongst patients enrolled on the Children’s Oncology Group (COG) B-ALL trial P9906 who were minimal residual disease (MRD) positive following one month of induction chemotherapy, those with low α4 integrin expression had significantly improved overall survival (OS) relative to those with high α4 integrin expression^[18]^. Another analysis of samples from children with relapsed B-ALL enrolled on the ALL-REZ Berlin-Frankfurt-Münster (BFM) 2002 trial from the BFM study group demonstrated that patients with increased VLA-4 expression at the time of first relapse had a significantly impaired response to chemotherapy and inferior event-free survival and OS^[19]^. The authors of this study went on to demonstrate that VLA-4 blocking antibodies attenuated the adhesion of B-ALL cells to BMSCs *in vitro*, and that this was sufficient to decrease cellular proliferation and overcome the cytarabine resistance conferred by co-culture with BMSCs^[19]^.

In healthy hematopoietic cells, VLA-4 demonstrates high affinity for vascular cell adhesion molecule-1 (VCAM-1), a cell surface protein that is highly expressed on the vascular endothelium, as well as the ECM glycoproteins fibronectin and osteopontin^[20]^. In particular, it is this affinity for VCAM-1 that is thought to play the predominant role in hematopoietic cell trafficking, as antibodies against VCAM-1 are sufficient to reduce progenitor cell adhesion to BMSCs both *in vitro*^[21]^ and *in vivo*^[22]^, while anti-fibronectin antibodies are not^[23]^. In the context of leukemia, the VLA-4/VCAM-1 interaction is similarly thought to be the primary mediator of chemoresistance. Co-culture of ALL cell lines with BMSCs, but not with BMSC conditioned media, significantly reduced sensitivity to cytarabine and etoposide, an effect that was abrogated in the presence of VCAM-1 blocking antibodies^[24]^.

Mechanistically, engagement of the VLA-4/VCAM-1 axis has been shown to activate pro-survival signaling pathways in ALL cells that in turn mediate chemoresistance. For example, *in vitro* stimulation of VLA-4 was shown to activate the PI3K/Akt signaling pathway in a B-ALL cell line, concomitant with the induction of adriamycin resistance, and inhibition of PI3K was sufficient to restore adriamycin sensitivity^[25]^. Furthermore, Astier *et al.*^[26]^ analyzed global gene expression changes in ALL cells following *in vitro* engagement of VLA-4 and found that a number of apoptotic regulators, including members of the caspase family, are significantly downregulated following VLA-4 activation while pro-survival factors, such as XIAP and survivin, are upregulated, together contributing to a chemoresistant state. Interestingly, there is also evidence to suggest that chemoresistance is mediated by reciprocal signaling through the VLA-4/VCAM-1 axis. Jacamo *et al.*^[27]^ demonstrated that co-culture of BMSCs with leukemic blasts resulted in an upregulation of NF-κB signaling in the BMSCs themselves, and that this was attenuated in the presence of a VLA-4 blocking antibody. Furthermore, genetic or chemical inhibition of NF-κB signaling in BMSCs restored sensitivity to vincristine in ALL cells both *in vitro* and *in vivo*. The authors speculate that the paracrine effects of many NF-κB target genes, including cytokines, that are induced in the presence of ALL cells may in turn facilitate the protective microenvironment provided by the BMSCs. Finally, VLA-4 may promote chemoresistance via its interactions with ion channels present at the leukemic cell surface. In particular, the potassium channel Kv11.1 has been shown to be overexpressed in ALL cell lines and patient samples^[28]^. Intriguingly, VLA-4 can form cell surface signaling complexes involving Kv11.1, that in turn lead to activation of ERK and PI3K/Akt signaling with the concomitant induction of chemoresistance^[29]^.

Given the well-established role of VLA-4 as a mediator of chemoresistance in ALL cells, there has been increasing interest in augmenting chemosensitivity through the use of anti-VLA-4 therapeutics, many of which have been used successfully in a number of autoimmune and inflammatory conditions due to their ability to modulate leukocyte activity. Natalizumab is a humanized monoclonal antibody that targets the α4 integrin subunit, thereby inhibiting VLA-4 and the closely related α4β7 integrin, which similarly binds VCAM-1^[30]^. Hsieh *et al.*^[18]^ demonstrated that natalizumab significantly prolonged survival in a xenograft model of B-ALL when combined with multi-agent chemotherapy consisting of vincristine, dexamethasone, and L-asparaginase relative to chemotherapy alone. Other therapeutic approaches involve peptide or non-peptide ligands that compete with VCAM-1 for VLA-4 binding. One such small molecule, TBC3486, has significantly increased affinity for VLA-4 relative to α4β7 and has been shown to enhance chemosensitivity *in vitro* in B-ALL cells co-cultured with BMSCs and *in vivo* in a xenograft model of B-ALL^[31]^.

In addition to VLA-4, several other integrins have been implicated in chemoresistance in ALL. For example, CD11b is an α integrin that is typically expressed on myeloid cells and plays an important role in cellular migration and extravasation during an immune response^[32]^. While expression in lymphoid cells is usually restricted to memory B-cells, Rhein *et al.*^[33]^ investigated the significance of aberrant CD11b expression on pre-B-ALL blasts. In this analysis, the authors demonstrated that high CD11b expression at diagnosis was an independent poor prognostic factor, with the CD11b-high patients demonstrating significantly higher rates of post-induction MRD relative to the CD11b-low patients. Furthermore, they demonstrated that the blasts remaining at the end of induction therapy had significantly higher CD11b expression relative to the cells analyzed prior to the initiation of therapy. Further studies are needed to assess the functional significance of increased CD11b expression on B-ALL cells. Finally, in T-ALL cell lines and patient samples, the collagen-binding α2β1 integrin has been shown to confer resistance to doxorubicin. Engagement of the α2β1 integrin with collagen resulted in increased activity of the MAPK signaling pathway and sustained expression of the anti-apoptotic protein Mcl-1. Consistent with these findings, doxorubicin sensitivity could be restored with MEK inhibition^[34]^.

### Cadherins

First described for their role in regulating epithelial-to-mesenchymal transitions during normal embryonic development, cadherins have since been recognized as a structurally and functionally diverse superfamily of transmembrane proteins that play important roles in normal cellular processes and in the development and progression of cancer^[35]^. The intracellular domains of cadherin proteins interact with a multitude of signal transduction effectors to carry out a variety of cellular processes. One crucial class of intracellular effectors is the catenin family of proteins, which provide a physical link between the intracellular domains of cadherin proteins and the actin cytoskeleton, thereby facilitating the role of cadherins in maintaining cell adhesion. In immune cells, the β-catenin protein, through its involvement in canonical Wnt pathway signaling, plays a critical role in the regulation of immune processes^[36]^.

Canonical Wnt signaling involves the binding of Wnt1 ligands to their receptor, known as Frizzled (Frz). In the absence of ligand binding, the intracellular β-catenin destruction complex is stabilized, resulting in low levels of intracellular β-catenin. Upon ligand binding, this complex becomes destabilized, allowing for an increase in levels of β-catenin protein in the cytoplasm. This β-catenin protein then translocates to the nucleus and associates with the TCF/LEF transcription factor complex, converting it from a repressive complex to an activating complex, which in turn activates a transcriptional program that mediates processes such as cell proliferation and differentiation^[37]^.

Of the cadherin subfamilies, the Fat cadherins are the most commonly mutated in ALL, and many reports have demonstrated their tumor suppressive activity. In an analysis of primary T-ALL samples from adult patients, Neumann *et al.*^[38]^ determined that 12% of the samples in their cohort had missense or nonsense mutations in *FAT1*, leading to loss of expression. Similar *FAT1* mutations have subsequently been reported in pediatric T-ALL^[4]^. Interestingly, loss of function mutations in *FAT1* have been associated with augmented Wnt pathway signaling. Specifically, Morris *et al.*^[39]^ demonstrated that the tumor suppressive effects of wild-type FAT1 protein derive from its ability to bind β-catenin at the periphery of the cell, thereby sequestering it in the cytoplasm and preventing its localization to the nucleus and activation of TCF/LEF complex. This aberrant activation of Wnt/β-catenin signaling has been implicated as a mediator of chemoresistance in ALL. Upon exposure of ALL cell lines and primary patient samples to BMSCs, Yang *et al.*^[40]^ found that the resulting cytarabine resistance was associated with inactivation of the β-catenin destruction complex and upregulation of members of the Wnt signaling pathway. Inhibition of β-catenin was sufficient to restore chemosensitivity both *in vitro* and *in vivo*. An integrated analysis of pairs of diagnostic and relapsed pediatric ALL samples incorporating gene expression, copy number alterations, and DNA methylation analyses revealed that dysregulation of this pathway was enriched at the time of disease relapse^[41]^, providing correlative evidence that altered Wnt/β-catenin signaling may be a clinically relevant mediator of resistance to chemotherapy. This same group went on to functionally test this interaction by demonstrating that, in contrast to their corresponding diagnostic samples, relapsed samples had significant overactivation of Wnt/β-catenin signaling and were highly refractory to the glucocorticoid prednisolone. In this context, the Wnt inhibitor iCRT14 synergized with prednisolone to induce apoptosis in relapsed patient samples^[42]^.

Intriguingly, other reports suggest that Fat cadherins may instead function as oncogenes, where they contribute to disease progression and chemoresistance. For example, de Bock *et al.*^[43]^ demonstrated that ALL cell lines express higher levels of FAT1 relative to healthy blood or bone marrow cells. The authors also demonstrated in two cohorts of pediatric patients with B-ALL that high FAT1 expression was associated with inferior relapse-free and OS. The same authors subsequently found that some T-ALL cell lines and patient samples express a truncated version of the FAT1 transcript, which in turn results in expression of a truncated protein that lacks the extracellular domain. Furthermore, this protein was found to cooperate with the Notch signaling pathway, described below, thereby serving an oncogenic function^[44]^.

### Galectins

The lectin family of cell surface proteins recognizes and binds to carbohydrates, leading to downstream signal transduction events that play crucial roles in normal physiology and in a variety of disease states^[45]^. The galectins represent a subclass of lectins with binding specificity for β-galactoside epitopes, commonly found in association with proteins that undergo glycosylation during their trafficking through the secretory pathway. As a result of their distinct affinities for specific glycoproteins, galectins provide a means of interpreting the information contained within these glycosylation events and converting that information into intracellular signal transduction processes. The ability of galectins to bind their glycoprotein ligands is facilitated in part by their ability to form higher order multimeric structures, thereby increasing their binding capacity^[46]^. In particular, galectin-3, which is commonly overexpressed in cancer, has extensive oligomerization capabilities, allowing it to form dynamic lattice structures at the cell surface that modulate the movement and activity of its glycoprotein binding partners^[47]^.

Galectin-3 was first identified as a potential mediator of chemoresistance in ALL cells through an analysis of gene expression changes following co-culture of ALL cells with BMSCs. Jurkat T-ALL cells were rendered resistant to doxorubicin upon co-culture with BMSCs, concomitant with upregulation of galectin-3. Forced overexpression of galectin-3 in these cells was sufficient to confer chemoresistance in the absence of BMSCs^[48]^. Consistent with these findings, Fei *et al.*^[49]^ found that *in vitro*, patient-derived B-ALL cells harvested from underneath a BMSC feeder cell layer, where they maintained direct cell-cell contact with feeder cells, had more galectin-3 on their cell surface relative to cells found in suspension above the cell layer or cells cultured in the absence of BMSCs. These same authors went on to assess secreted galectin-3 levels in the growth medium from BMSCs cultured in the absence or presence of ALL cells, and found that BMSCs secreted more galectin-3 in the context of ALL cell co-culture relative to culture of BMSCs alone, while ALL cells alone did not secrete galectin-3 under any conditions. These results suggested that the elevated galectin-3 found on the cell surface of ALL cells had a stromal origin. The authors further confirmed this by demonstrating that exosomes derived from BMSCs, but not from ALL cells, contained galectin-3 and that these exosomes were secreted by BMSCs and subsequently taken up by ALL cells^[50]^. Hu *et al.*^[50]^ similarly demonstrated that co-culture of ALL cells with BMSCs resulted in increased cell surface galectin-3 and chemoresistance, and extended this finding to demonstrate that transcriptional targets of the Wnt/β-catenin signaling pathway, previously implicated in chemoresistance, were upregulated in wild-type ALL cells co-cultured with BMSCs but not in galectin-3 knockout cells, suggesting that activation of this pathway is galectin-3-dependent^[51]^. Interestingly, galectin-3 inhibitors have demonstrated efficacy in acute myeloid leukemia^[52]^, diffuse large B-cell lymphoma^[53]^, and multiple myeloma^[54,55]^. Additional studies are necessary to determine whether such inhibitors may similarly augment chemosensitivity in the context of ALL.

### Notch signaling

The Notch signal transduction pathway is unique in several ways. First, its activation is dependent upon direct cell-cell contact between a ligand-expressing cell, such as a T-cell, and a receptor-expressing cell, such as a BMSC. Second, unlike other non-enzymatic transmembrane receptors, upon engagement of the Notch receptor by its ligands Delta-like or Jagged, the receptor itself undergoes proteolytic cleavage. Specifically, the extracellular domain of the receptor is shed first, followed by cleavage within the transmembrane domain by the γ-secretase complex. This allows the intracellular domain of the receptor (NICD) to translocate to the nucleus and function as a transcriptional regulator^[56]^.

In normal physiology, Notch signaling plays a critical role in T- and B-cell development. In T-cells, Notch activity is required for cell survival and proliferation during the double negative (DN) stages of thymocyte development, and is important for β selection during the production of a fully rearranged αβ T-cell receptor (TCR)^[57]^. Further highlighting the importance of Notch signaling for thymocyte development, ectopic expression of Delta-like 1 in the OP9 stromal cell line is sufficient to support early T-cell differentiation *ex vivo*^[58]^. Importantly, aberrant Notch signaling is a common feature of T-ALL. In pediatric T-ALL, activating mutations in components of the Notch signaling pathway, including in *NOTCH1* itself, are found in nearly 80% of diagnostic samples^[4]^. Though not found to be mutationally activated in B-ALL, Notch signaling does also play an important role in B-cell development where it promotes the differentiation of marginal zone and follicular zone B-cells^[59]^.

Interestingly, within the bone marrow microenvironment, Notch signaling has been shown to mediate chemoresistance in the setting of hypoxia. Specifically, the bone marrow is a highly hypoxic environment, with a reported oxygen saturation of only 87.5% in healthy subjects, compared to 99% in peripheral blood^[60]^. Upon exposure to a hypoxic environment, cells upregulate expression of the transcription factor hypoxia-inducible factor-1α (HIF-1α), which mediates a variety of tissue-specific transcriptional programs^[61]^. In solid tumor cells, it has been shown that Notch signaling is induced in response to hypoxia and functions to promote cellular motility and invasiveness^[62]^. Zou *et al.*^[63]^ similarly demonstrated that in the setting of hypoxia, T-ALL cells undergo HIF-1α-dependent Notch activation. In T-ALL cell lines, exposure to hypoxia was sufficient to confer chemoresistance, and this could be overcome with Notch silencing.

Due to the role for Notch signaling as a mediator of chemoresistance, there is interest in pharmacologically targeting the Notch signaling pathway. In addition to monoclonal antibodies targeting Notch receptors or ligands^[64]^, significant attention has been devoted to the preclinical and clinical development of γ secretase inhibitors (GSIs). Interestingly, numerous preclinical studies have demonstrated the ability of GSIs to augment chemosensitivity^[65-67]^. Unfortunately, early single agent clinical trials involving GSIs were limited by severe gastrointestinal toxicity^[68]^. However, Real *et al.*^[69]^ demonstrated through a series of elegant *in vivo* experiments that GSIs effectively overcome glucocorticoid resistance in T-ALL, and that concomitant exposure to glucocorticoids and GSIs abrogates the gastrointestinal toxicity associated with GSIs, thereby restoring the potential for the use of GSIs to modulate response to chemotherapy in T-ALL. This strategy was employed in a clinical trial involving relapsed or refractory pediatric solid tumors and T-cell leukemia (NCT01088763).

## Soluble factors

In addition to direct cell-cell interactions, a number of secretory products made by cells within the bone marrow microenvironment engage with receptors on lymphoid cells to mediate functions such as cell proliferation and survival. Like the factors described above, many of these soluble products have similarly been shown to mediate chemoresistance.

### TNFα

TNFα is a pro-inflammatory cytokine that is responsible for normal immune system homeostasis and plays numerous well-established roles in the development and maintenance of malignancy^[70]^. Produced by macrophages, natural killer cells, and T-cells, TNFα normally functions as a regulator of hematopoiesis when present in conjunction with other growth factors that similarly act to maintain the appropriate size and function of the hematopoietic compartment^[71]^. Interestingly, a polymorphism in the *TNF* gene itself, which results in higher plasma levels of TNFα, has been shown to corelate with poor outcomes in a number of hematologic malignancies, including Hodgkin’s^[72]^ and non-Hodgkin’s lymphoma^[73]^. Consistent with these findings, Lauten *et al.*^[74]^ demonstrated in a cohort of children treated on BFM protocols that amongst patients with a prednisone poor response, those with TNF gene polymorphisms had a higher rate of relapse. Mechanistically, TNFRII signaling has been shown to activate the PI3K/Akt signal transduction pathway, leading to downstream pro-survival signaling. Activation of this pathway has been associated with the induction of doxorubicin resistance in ALL cell lines^[75]^, though further studies are needed to fully elucidate the relationship between altered TNFα expression and signaling and clinical outcomes for patients with ALL.

### γ chain cytokines

Consisting of interleukin (IL)-2, 4, 7, 9, 15, and 21, the common γ chain family of cytokines exert their effects on immune cells via a receptor that contains one or more cytokine-specific receptor chains as well as the common cytokine receptor γ chain. These multimeric receptor complexes lack intrinsic enzymatic activity and instead recruit downstream effectors via their cytoplasmic domains. Specifically, the cytokine-specific receptor chains most commonly recruit the kinase JAK1, while the γ chain recruits JAK3. Activation of these JAK proteins results in phosphorylation of the cytoplasmic domains of the receptor subunits, which in turn creates docking sites for the STAT family of transcription factors, with STAT protein binding specificity determined by the identity of the specific receptor docking sites. Upon recruitment to activated cytokine receptors, these STAT proteins undergo JAK-mediated phosphorylation, leading to their translocation to the nucleus where they function as transcription factors to mediate target gene expression^[76]^. Due to their central roles in regulating the differentiation, survival, and proliferation of healthy lymphoid cells, it is not surprising that γ chain cytokine receptors play an important role in mediating chemoresistance in ALL.

Produced by T-cells, IL-4 acts on a multitude of immune cell subtypes leading to a wide range of downstream effects. For example, IL-4 is a signature cytokine in the differentiation of naïve CD4+ T-cells into the Th2 subtype of T helper cells. In the B-cell lineage, IL-4 functions as a B-cell differentiation factor, promoting immunoglobulin isotype switching, and in the myeloid lineage, IL-4 is important for the development of M2 macrophages^[76]^. Importantly, IL-4 has been implicated in glucocorticoid resistance in both non-malignant^[77,78]^ and malignant T-cells. Serafin *et al.*^[79]^ discovered that in T-ALL patients with a poor initial response to the glucocorticoid prednisone, the *IL4* gene was upregulated downstream of aberrant activation of LCK, a Src family non-receptor tyrosine kinase that is important for lymphocyte development and activation. The authors demonstrated that culturing glucocorticoid sensitive T-ALL cell lines in the presence of IL-4 was sufficient to confer glucocorticoid resistance. Interestingly, in conjunction with TNFα, IL-4 is also a potent inducer of VCAM-1 expression^[80]^, with TNFα and IL-4 exposure promoting the adhesion of ALL cells to bone marrow fibroblasts in culture^[21,81]^. As described above, the VLA-4/VCAM-1 axis has been implicated in chemoresistance in a variety of model systems.

IL-7 is produced by the stromal cells of the bone marrow and thymus and plays a crucial nonredundant role in lymphoid cell development and survival. Activation of the IL-7 receptor (IL-7R) primarily recruits the STAT5 transcription factors, which induce expression of antiapoptotic proteins such as BCL-2. Interestingly, many studies of early thymocyte development have demonstrated an interaction between IL-7R signaling, TCR signaling, and endogenous glucocorticoid exposure that is critical for the appropriate development of the T-cell repertoire. Specifically, in normal T-cell development, IL-7R is expressed at high levels in the early DN thymocyte population, thereby maintaining cell survival prior to rearrangement of the TCR^[76]^. Upon induction of TCR rearrangement during the late DN and early double positive (DP) stage of development, IL-7R is transiently downregulated, thereby abrogating its strong pro-survival signal^[82]^. At this stage, exposure to endogenous glucocorticoids is sufficient to induce cell death unless the cell is rescued by pro-survival signaling following successful TCR rearrangement^[83]^. In this model, loss of IL-7R-mediated pro-survival signaling is required to enable an apoptotic response to endogenous glucocorticoids in the absence of an appropriately rearranged TCR. In the context of T-ALL, signaling through the IL-7R/JAK/STAT5 pathway has been shown to confer resistance to pharmacologic concentrations of glucocorticoids, suggesting that this normal developmental process is co-opted in this malignant state to promote chemotherapy resistance.

Specifically, gain-of-function mutations in the IL-7R/JAK/STAT5 pathway occur in 25% of pediatric and young adult T-ALLs^[4]^, underscoring the strong selective pressure for activation of this pathway as a mediator of T-cell survival. Both mutational and non-mutational activation of this pathway have been implicated in glucocorticoid resistance in T-ALL. For example, Li *et al.*^[10]^ demonstrated in diagnostic samples from children with T-ALL that mutations in the IL-7R/JAK/STAT5 pathway were associated with decreased *ex vivo* prednisolone sensitivity and with inferior relapse free survival. The authors went on to express mutant alleles of IL-7R pathway genes in glucocorticoid sensitive T-ALL cell lines and found that many of these mutations were sufficient to confer resistance to glucocorticoids, but not to other chemotherapies. Consistent with this idea, we previously demonstrated that IL-7-induced glucocorticoid resistance most commonly occurs in T-ALLs that lack activating mutations in the IL-7R/JAK/STAT5 pathway, and that this resistance phenotype is enriched in T-ALLs of the early T-cell precursor (ETP) subset, which correspond to the early DN stages of thymocyte development. Like Li *et al.*^[10]^, we similarly found that IL-7-mediated effects on chemoresistance are specific to glucocorticoids^[84]^. We later showed that glucocorticoid resistance is mediated specifically by induction of the anti-apoptotic protein BCL-2 downstream of STAT5, and can be overcome with inhibition of JAK. Interestingly, we further demonstrated that this mechanism of IL-7-induced glucocorticoid resistance occurs in distinct subpopulations of healthy developing thymocytes both *in vivo* and *ex vivo*, and that T-ALLs with gene expression signatures resembling early stages of thymocyte development are enriched for this resistance phenotype^[85]^. Taken together, these data provide strong evidence for a developmentally-retained mechanism by which T-ALLs arising from stages of thymocyte development that rely on IL-7R signaling can utilize this pathway to resist apoptosis in response to glucocorticoid therapy even in the absence of activating pathway mutations.

While not a γ chain cytokine receptor, the thymic stromal lymphopoietin (TSLP) receptor (TSLP-R) consists of a heterodimer of the IL-7Rα chain and cytokine receptor-like factor 2 (CRLF2), and plays a role in normal B-cell development^[86]^. Aberrant signaling through TSLP-R, most commonly mediated by chromosomal rearrangements of *CRLF2* and/or mutational activation of its downstream effector, *JAK2*, is a hallmark feature of Philadelphia chromosome-like (Ph-like) B-ALL. These leukemias demonstrate a gene expression signature that is similar to that of Ph-positive B-ALL but lack the BCR-ABL1 translocation^[87]^. Importantly, *CRLF2* rearrangements and *JAK2* mutations, collectively leading to increased TSLP-R signaling capacity, have been associated with poor outcomes in patients with Ph-like ALL^[88]^. Similar to T-ALL, we previously demonstrated that *CRLF2*-rearranged Ph-like B-ALL samples uniformly demonstrate *in vitro* glucocorticoid resistance^[89]^.

Given the role of cytokine-mediated JAK/STAT signaling in the pathogenesis and chemoresistance of ALL cells, there has been considerable interest in targeting this pathway as a means of improving clinical outcomes. While several approaches have been studied, including the use of reducing agents to disrupt disulfide bond formation within IL-7R, blocking antibodies against IL-7R, and IL-7R-directed chimeric antigen receptor (CAR) T-cells^[90]^, small molecule inhibitors of JAK are currently furthest along in clinical development. Ruxolitinib is a JAK1/2 inhibitor that has shown efficacy as a single agent in xenograft models of ETP T-ALL^[91]^ and Ph-like ALL^[92]^, and has been shown to sensitize ALL cells to glucocorticoids *in vitro*^[84,85,89]^. Consistent with these promising results, clinical trials have been initiated to investigate the efficacy of ruxolitinib in ALL (NCT03571321 and NCT02723994).

### Chemokines

Chemokines are a subclass of cytokines that are specifically responsible for the export of immune cells from the bone marrow and for the homing of these cells to sites of infection and inflammation. Chemokine ligands characteristically contain one or more cysteine residues, enabling their division into the XC, CC, CXC, CX3C subgroups on the basis of the location of their cysteine residues within their amino acid sequences. Upon secretion, chemokines bind to G-protein-coupled receptors, with some receptors showing specificity for a single ligand and others capable of binding multiple distinct ligands^[93]^. In particular, CXCR4 signaling has been implicated in the initiation and progression of ALL, and has further been shown to mediate chemoresistance.

CXCL12, also called stromal derived factor 1 (SDF-1) is the chemokine ligand for the CXCR4 receptor and is expressed ubiquitously at all stages of development, where it plays an important role in tissue homeostatic functions, including within the hematopoietic compartment^[94]^. Importantly, aberrant CXCL12/CXCR4 signaling has been functionally implicated in leukemogenesis and associated with poor clinical outcomes. For example, it has been shown that the protein phosphatase calcineurin is aberrantly activated in T-ALL and is required for the activity of leukemia initiating cells^[95]^. Passaro *et al.*^[96]^ further demonstrated that cell surface expression of CXCR4 is regulated by calcineurin activity, and the presence of CXCR4 at the cell surface is required for the migratory capability of T-ALL cells. The authors further showed that silencing of CXCR4 was associated with the induction of apoptosis in a T-ALL cell line and in a patient-derived xenograft model of T-ALL. In pediatric B-ALL, high CXCR4 expression was associated with inferior relapse free survival^[97]^, and in adult B-ALL, phosphorylated CXCR4, corresponding to an active form of the receptor, was associated with reduced OS^[98]^.

To assess the relationship between CXCR4 expression and chemosensitivity in ALL, Sison *et al.*^[99]^ utilized primary samples from infants with mixed lineage leukemia (*MLL*)-rearranged ALL, a high-risk molecular subtype of B-ALL that is associated with poor outcomes. Consistent with other reports, the authors found that exposure to BMSCs conferred chemoresistance and that co-treatment with the CXCR4 antagonist plerixafor was sufficient to restore chemosensitivity. The same group went on to demonstrate that in response to chemotherapy, ALL cells upregulate expression of CXCR4, priming them to respond to BMSC-secreted CXCL12. In light of these findings, the authors demonstrated the efficacy of a combinatorial therapeutic strategy involving plerixafor in an *in vivo* xenograft model and found that co-treatment with plerixafor abrogated the protective effect of the bone marrow microenvironment on the response to chemotherapy^[100]^. As a result of these promising results in preclinical models, the Pediatric Oncology Experimental Therapeutics Investigators’ consortium conducted a phase 1 study to evaluate the potential for combining plerixafor with the chemotherapies cytarabine and etoposide in children with relapsed or refractory acute leukemia (NCT01319864). While the only clinical responses observed in this cohort occurred in children with AML, the number of patients with ALL was small^[101]^. Based on these results, the role of plerixafor in augmenting chemosensitivity remains to be studied in additional patient cohorts in conjunction with other chemotherapy backbones.

### Asparagine

The introduction of L-asparaginase into modern treatment regimens has dramatically improved outcomes for patients with ALL^[102]^. The efficacy of L-asparaginase relies upon the unique requirement for asparagine in leukemia cells. In healthy cells, asparagine is a non-essential amino acid, synthesized intracellularly through the activity of asparagine synthetase. In contrast, ALL cells lack sufficient expression of asparagine synthetase to meet their requirement for asparagine, thereby relying instead on exogenous sources. The use of L-asparaginase exploits this dependency by hydrolyzing asparagine, thus depriving ALL cells of this exogenous supply^[103]^. BMSCs have been shown to contribute to L-asparaginase resistance through upregulation of asparagine synthetase. Specifically, Iwamoto *et al.*^[104]^ demonstrated that expression of the gene encoding asparagine synthetase, *ASNS*, was significantly higher in BMSCs than in ALL cells themselves, and that co-culture of ALL cells with BMSCs conferred resistance to L-asparaginase, suggesting that BMSCs function as an exogenous source of asparagine for ALL cells.

## Conclusion

Comprehensive analyses of the genomic and transcriptomic landscape of ALL have revealed many factors that contribute to chemoresistance and poor clinical outcomes for patients with ALL. In contrast to these cell-intrinsic drivers of chemoresistance, cell-extrinsic factors are less well-appreciated for their role in modulating clinical outcomes. As described here, the bone marrow microenvironment, through direct cell-cell interactions and the production of soluble factors, plays a complex and multifactorial role in determining the response of ALL cells to chemotherapy. This review highlights a number of efforts to target the microenvironment as a means of augmenting the efficacy of chemotherapy. While many of the preclinical studies described here present promising results, further studies are needed to validate these therapeutic strategies in other preclinical model systems and to facilitate their eventual clinical implementation.

## Declarations

### Copyright

© The Author(s) 2019.
